# Hyperglycemia Promotes TMPRSS2-ERG Gene Fusion in Prostate Cancer Cells *via* Upregulating Insulin-Like Growth Factor-Binding Protein-2

**DOI:** 10.3389/fendo.2017.00305

**Published:** 2017-11-06

**Authors:** Jeff M. P. Holly, Jessica Broadhurst, Rehanna Mansor, Amit Bahl, Claire M. Perks

**Affiliations:** ^1^IGFs & Metabolic Endocrinology Group, School of Clinical Sciences, Southmead Hospital, Bristol, United Kingdom; ^2^Department of Clinical Oncology, Bristol Haematology and Oncology Centre, University Hospitals Bristol, Bristol, United Kingdom

**Keywords:** prostate cancer, insulin-like growth factor-binding protein-2, TMPRSS2-ERG, hyperglycemia, type II diabetes

## Abstract

**Background:**

Epidemiologic evidence shows that obesity is associated with a greater risk of aggressive prostate cancer (PCa) and PCa-specific mortality and this is observed mainly in men with the *TMPRSS2-ERG* gene fusion. Obesity is often associated with comorbid conditions such as type 2 diabetes and hyperglycemia: we investigated whether some of the exposures associated with disturbed metabolism can also affect the frequency of this gene fusion.

**Methods:**

Fusion was induced in LNCaP PCa cells in normal or high levels of glucose, with or without insulin-like growth factor binding protein-2 (IGFBP-2) silenced or the presence of insulin-like growth factor-1 (IGF-I), insulin, or epidermal growth factor (EGF). RNA was extracted for analysis by nested PCR. Abundance of IGFBP-2, γH2AX, DNA-dependent protein kinase catalytic subunit (DNAPKcs), and β-actin were analyzed by Western immunoblotting.

**Results:**

Our data suggest that hyperglycemia-induced IGFBP-2 increased the frequency of the gene fusion that was accompanied by decreased levels of DNAPKcs implying that they were mediated by alterations in the rate of repair of double-strand breaks. In contrast insulin, IGF-I and EGF all decreased gene fusion events.

**Conclusion:**

These novel observations may represent a further mechanism by which obesity can exert an effect aggravating PCa progression.

## Introduction

The *TMPRSS2-ERG* fusion oncogene is thought to be important during tumor progression and development as it is found in approximately half of all prostate cancer (PCa) biopsies and also in metastases (1–3).

Joining of the 5′-untranslated region of *TMPRSS2* with the oncogenic *ETS* transcription factor, ERG culminates in the *TMPRSS2-ERG* gene fusion. TMPRSS2 possesses androgen-responsive elements and so in response to androgens *TMPRSS2* drives *ERG* overexpression. Antiandrogens can decrease *ERG* in patients carrying *TMPRSS2-ERG* through its ability to reduce the levels of androgen. In contrast, for patients whose PCa progresses and becomes resistant to antihormone therapy, the fusion oncogene*TMPRSS2-ERG* can be reactivated and could thus contribute to tumor progression (4).

We are currently facing a global obesity epidemic that has been associated with a negative impact on PCa. There is strong epidemiologic evidence that obesity is associated with a greater risk of aggressive PCa and increased PCa-specific mortality (5–7). Furthermore, the negative impact of obesity on PCa prognosis has mainly been observed in men with the *TMPRSS2-ERG* gene fusion (8) implying an interaction.

Obesity is often associated with comorbid conditions such as insulin resistance, hyperglycemia, and type 2 diabetes. We have shown previously that hyperglycemia-induced chemoresistance in PCa cells and that this was mediated by an epigenetic upregulation of insulin-like growth factor binding protein-2 (IGFBP-2) (9). IGFBP-2 is one of the six high-affinity IGF-binding proteins, which bind to IGFs, acting as a carrier and protecting them from clearance, increasing their half-lives, and modulating their availability and activity. These IGFBPs, including IGFBP-2 can also regulate cell function independently of the insulin-like growth factor-1 (IGF-I) receptor (10, 11). IGFBP-2 is considered to be a key player in PCa progression (12) with IGFBP-2 levels being raised in the serum and in the tumors of patients with PCa (13, 14).

PTEN is a phosphoprotein that exhibits both protein- and lipid-phosphatase activity that inhibits the phosphatidylinositol 3-kinase/Akt and mitogen-activated protein kinase signaling pathways (15–17), thereby acting in an opposite manner to growth factors, which promote cell growth and survival. We identified that IGFBP-2 inhibited PTEN function in PCa cells by increasing its phosphorylation (18) and global expression profiling indicated that IGFBP-2 was the most important biomarker to indicate the status of PTEN in tumors (19). When PTEN is silenced, mice develop high grade prostatic intraepithelial neoplasia, but do not progress to develop cancer (20). 93% of ERG rearrangement positive samples showed either absent or reduced PTEN (21) and tumors lacking functional PTEN express higher levels of ERG rearrangement (22). IGFBP-2 has also been shown to translocate to the nucleus in neuroblastoma cells, *via* its nuclear localization sequence, where it directly associates with DNA and functions as a transcription factor, modulating specific tumorigenic genes (23, 24).

The high frequency of the TMPRSS2-ERG gene fusion in PCa is not due to random translocations but is promoted by the androgen receptor inducing changes in chromosomal architecture leading to the proximity of the TMPRSS2 and ERG genes that are then fused following double-strand breaks (DSB) and repair *via* the non-homologous end joining (NHEJ) pathway (25). There have been several studies examining the effects of androgen exposure on the formation of fusion products (26, 27), but little work examining potential effects of other exposures. In this study, we examined the effect of some of the exposures associated with disturbed metabolism on *TMPRSS2-ERG* gene fusion, in particular, hyperglycemia and the potential role of IGFBP-2 in the latter.

## Materials and Methods

### Materials

All chemicals, unless otherwise stated, were purchased from Sigma (Poole, UK). LNCaP cells were bought from ATCC and cultured as described previously (9). All cell lines tested negative for mycoplasma.

### Fusion Induction

LNCaP cells were seeded in 6-well plates in DMEM growth media (Basel, Switzerland, GM: 5 mM glucose) with or without IGFBP-2 silenced for 24 h, serum starved for 24 h in the presence of aphidicolin (2 µg/ml) in DMEM and HAM’S Nutrient Mix F12 media containing charcoal-stripped serum (Invitrogen, Paisley, UK, SFM: 25 mM) and then dosed with dihydrotestosterone (DHT:0.1 μM) in the presence or absence of IGF-I, Gropep, Adelaide, SA, Australia (100 ng/ml), insulin, Novo Nordisk, West Sussex, UK (100 ng/ml), or epidermal growth factor (EGF), Merck, Hertfordshire, UK (20 ng/ml) for 2 h in fresh charcoal-stripped serum based media followed by the addition of etoposide (60 µM) for 1 h. We confirmed that dosing with etoposide at this dose for 1 h did not induce any consequent cell death (data not shown). Before assessing whether IGF-I, insulin, or EGF affected the rate of fusion in LNCaP cells, we initially assessed how responsive these cells were to the factors in relation to DNA proliferation. On performing dose responses, we found that 20 ng/ml EGF and 100 ng/ml IGF-I and insulin gave the greatest response in terms of growth and so used these doses for the fusion experiments. Optimum doses of DHT and etoposide that were used were selected from previous dose response curves (data not shown). Cells were incubated in fresh charcoal-stripped serum based media for a further 24 h prior to the extraction of RNA using Trizol reagent from Invitrogen (Carlsbad, CA, USA) according to manufacturer’s instructions and conversion to cDNA using a kit from Invitrogen (SuperScript III First-Strand Synthesis System). IGFBP-2 was silenced, parallel to non-silencing controls, using siRNA (100 nM) and a second siRNA for IGFBP-2 was also used to exclude off-target responses: sequences of siRNAs and methodology described previously (9, 28).

### Quantitative Nested PCR

Each tube of cDNA was separated into 10 × 2 µl aliquots. These were used in 10 separate nested PCR reactions amplified using primer pair 1 (TMPRSS2 forward CAGGAGGCGGAGGCGGA: ERG reverse GGCGTTGTAGCTGGGGGTGAG). 2 μl of this PCR product was then taken and used to initiate the second round of PCR amplified using primer pair 2 (TMPRSS2 forward GGAGCGCCGCCTGGAG: ERG reverse CCATATTCTTTCACCGCCCACTCC) in a further 10 reactions as described previously (29). Each PCR product was run on a 1.7% agarose gel and the total number of PCR products from these 10 reactions counted and compared. This process was repeated for each treatment in triplicate.

### Western Immunoblotting

Insulin-like growth factor binding protein-2, γH2AX, DNA-dependent protein kinase (DNAPK)cs, and β-actin were analyzed by Western immunoblotting as described previously (9).

### Statistical Analysis

Data were analyzed with SPSS 12.0.1 for Windows using one-way ANOVA followed by least significant difference *post hoc* test. A statistically significant difference was present at *p < 0.05.

## Results

### Effect of Glucose on the Number of TMPRSS2:ERG Fusion Products: A Role for IGFBP-2

**Figure 1:**
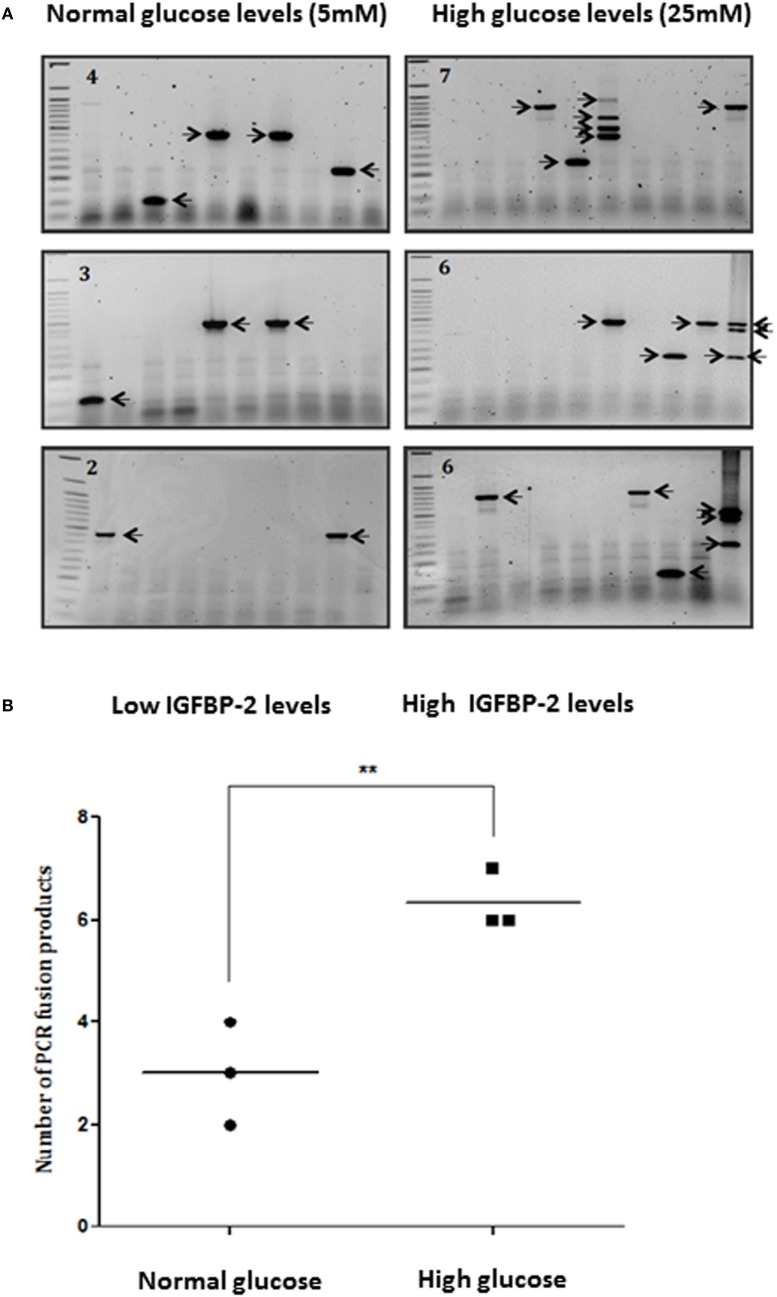

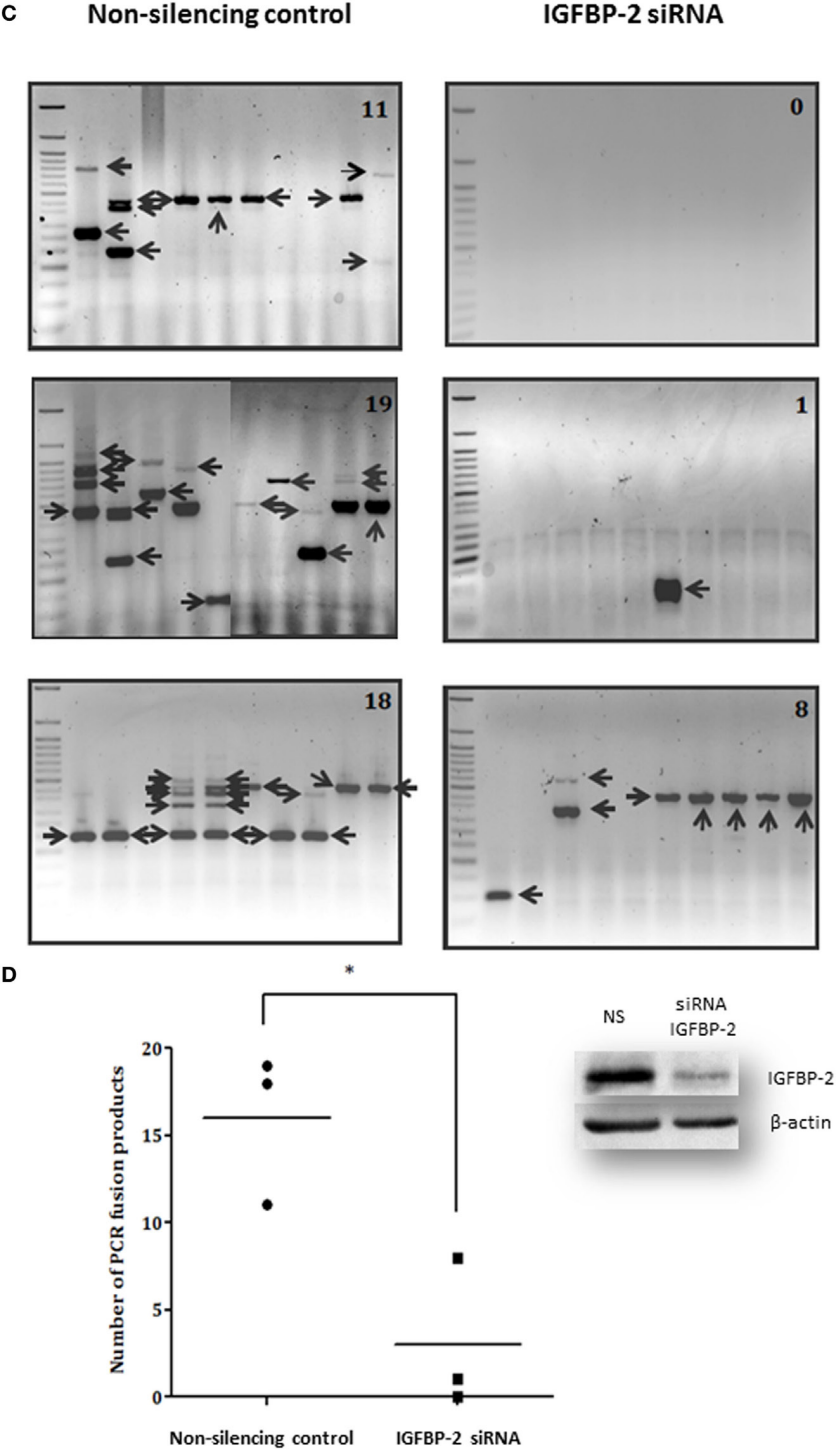
Effects of hyperglycemia on TMPRSS2:ERG fusion induction in LNCaP prostate cancer cells. LNCaP cells were seeded (0.7 × 10^6^ cells/well) in 6-well plates in normal glucose-containing DMEM growth media (GM: 5 mM glucose) for 24 h, serum starved for 24 h in the presence of aphidicolin (2 µg/ml) in either normal or high glucose-containing DMEM and HAM’S Nutrient Mix F12 media containing charcoal-stripped serum (SFM: 25 mM), and then dosed with DHT (0.1 µM) for 2 h in fresh charcoal-stripped serum based media followed by the addition of etoposide (60 µM) for 1 h. Cells were incubated in fresh charcoal-stripped serum based media for a further 24 h. LNCaP cells were also seeded (0.7 × 10^6^ cells/well) in high glucose-containing GM and insulin-like growth factor binding protein-2 (IGFBP-2) was silenced parallel to non-silencing controls using siRNA (100 nM). After 16 h cells were serum starved in high glucose-containing SFM as above for a further 24 h and treated with 0.1 µM DHT for 2 h and 60 µM etoposide for a further 1 h. Cells were incubated in fresh SFM for a further 24 h. Cells were extracted in trizol for performing nested PCR. **(A,C)** Illustrate three repeats of the gels showing PCR products (each indicated by an arrow) and number of PCR products in top corner of each blot **(B,D)** is the quantification of the PCR products. Insert to **(D)** is a representative western immunoblot for IGFBP-2 and β-actin (NS, non-silencing siRNA).

Figures 1A,B show that the average number of TMPRSS2:ERG fusion products was higher in 25 mM glucose (6.3) compared to 5 mM glucose (3), with the average rate over 2.1-fold higher at 25 mM than at 5 mM (*p* < 0.01). As we have shown previously, using ELISA and western blotting that high glucose increases the abundance of IGFBP-2 compared with levels observed in 5 mM glucose by 1.8-fold (*p* < 0.01) (9), these data suggested that the glucose-induced increase in IGFBP-2 may be related to the increase in TMPRSS2:ERG fusion products. Therefore, to examine this more specifically, we silenced IGFBP-2 using siRNA in high glucose conditions and observed a significant decrease in TMPRSS2:ERG fusion products (*p* < 0.05): effective silencing of IGFBP-2 is indicated by a western blot (Figures 1C,D).

### Effect of Insulin, EGF, and IGF-I on the Number of TMPRSS2-ERG Fusion Products

**Figure 2:**
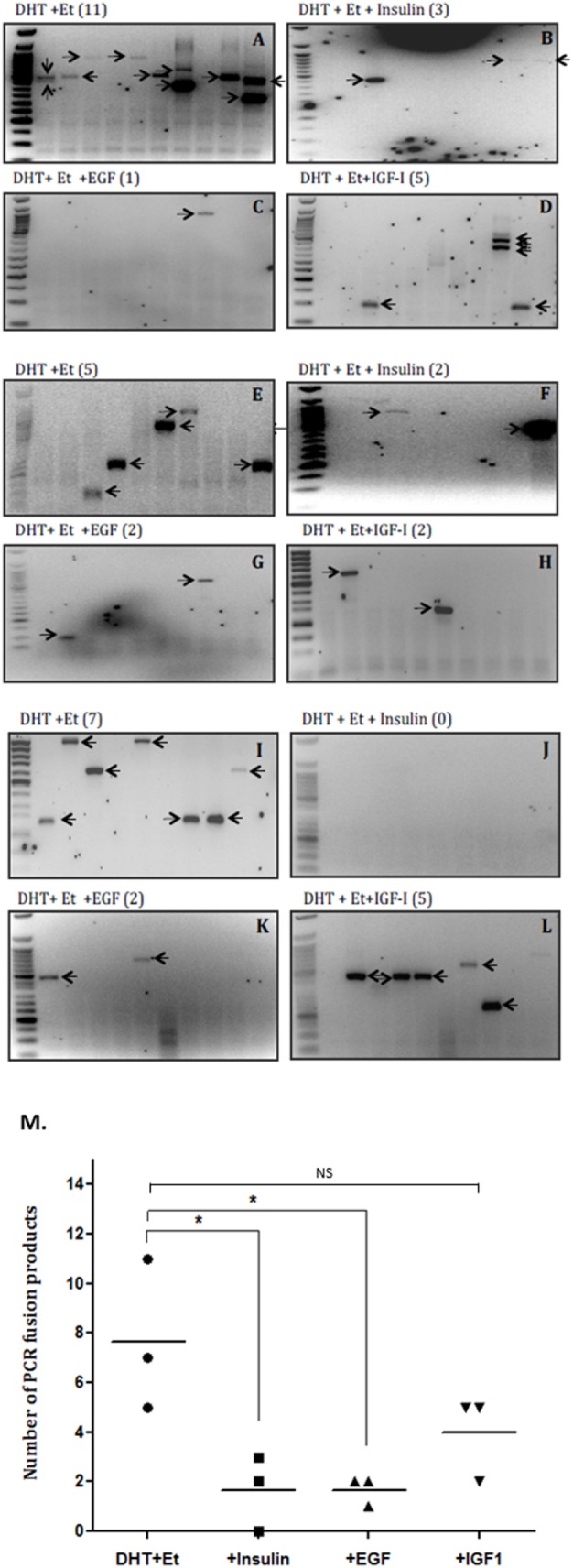
Effects of insulin, epidermal growth factor (EGF), and insulin-like growth factor-1 (IGF-I) on TMPRSS2:ERG fusion induction in LNCaP prostate cancer cells. LNCaP cells (0.7 × 10^6^ cells/well) were seeded in 6-well plates and serum starved as described in figure legend 1 for a further 24 h and treated with 0.1 µM DHT for 2 h in the presence or absence of IGF-I (100 ng/ml), insulin (100 ng/ml), or EGF (20 ng/ml) followed by 60 µM etoposide for a further hour. A final 24 h incubation with fresh SFM was followed by RNA extraction in Trizol for analysis by nested PCR. **(A–L)** Illustrates three repeats of the gels showing PCR products with the number of PCR products in brackets after the title of each blot and **(M)** is the quantification of the PCR products.

The average rate of fusion induction was decreased 3.5-fold by insulin (that does not bind IGFBPs) (Figures 2A,B,E,F,I,J) and over 2.5-fold by IGF-I (Figures 2A,D,E,H,I,L). We also observed a 3.5-fold decrease by an alternative growth factor, EGF (Figures 2A,C,E,G,I,K). While the decrease in fusion induction with insulin and EGF treatment were statistically significant (*p* < 0.05), the decrease observed in the presence of IGF-I was not statistically significant (Figure 2M).

### Effects of Silencing IGFBP-2 or Adding IGF-I on Levels of γH2AX and DNA-Dependent Protein Kinase Catalytic Subunit (DNAPKcs)

**Figure 3:**
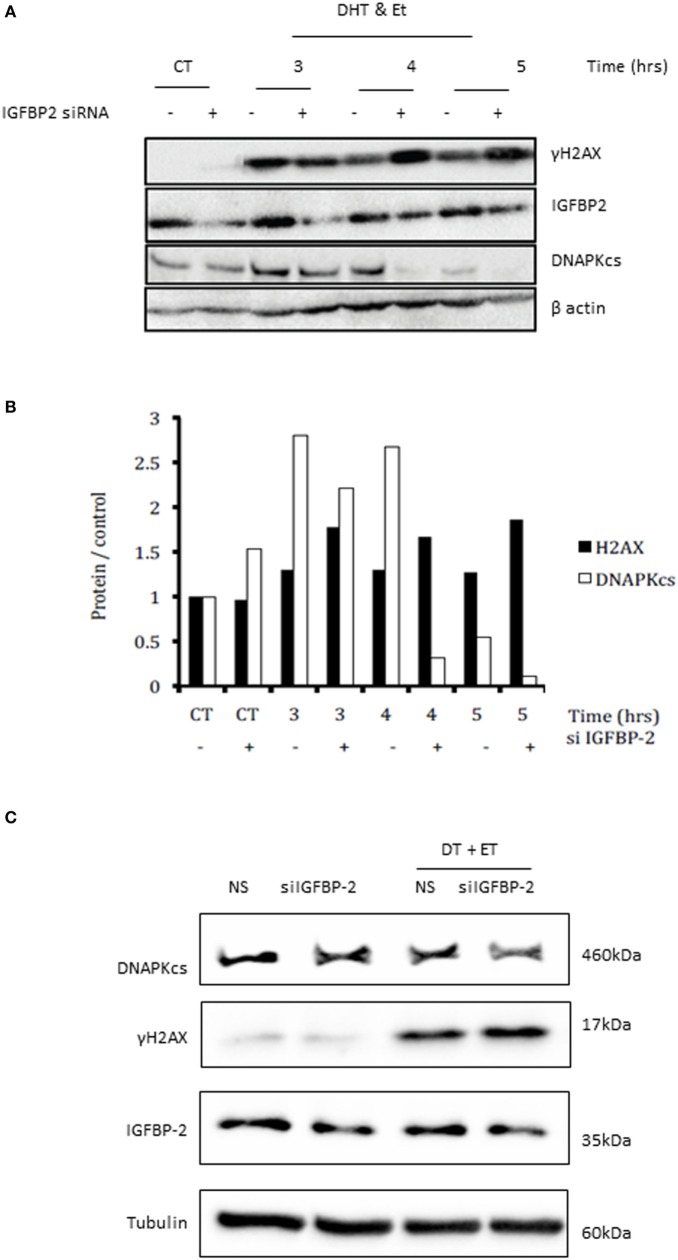

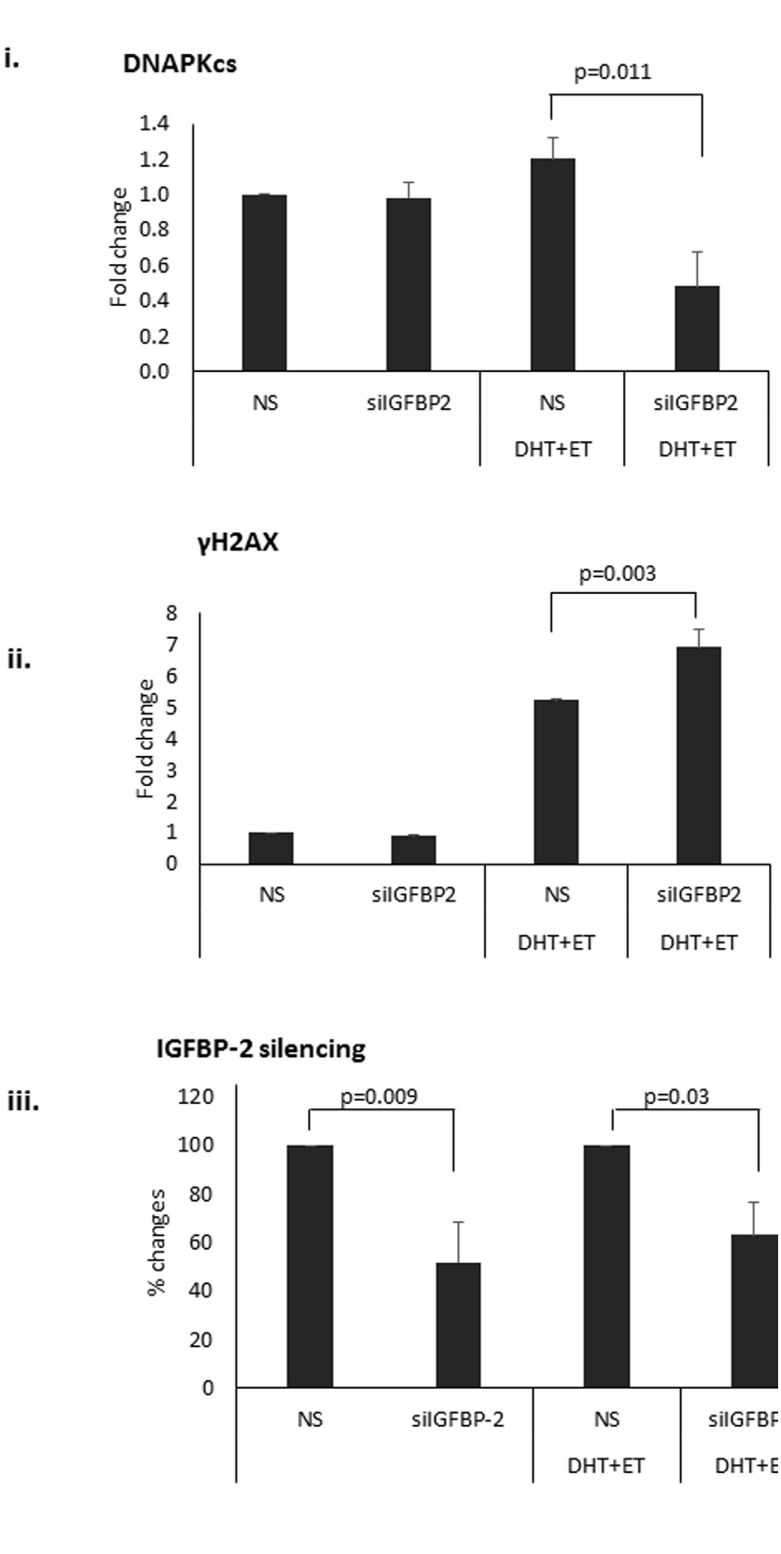

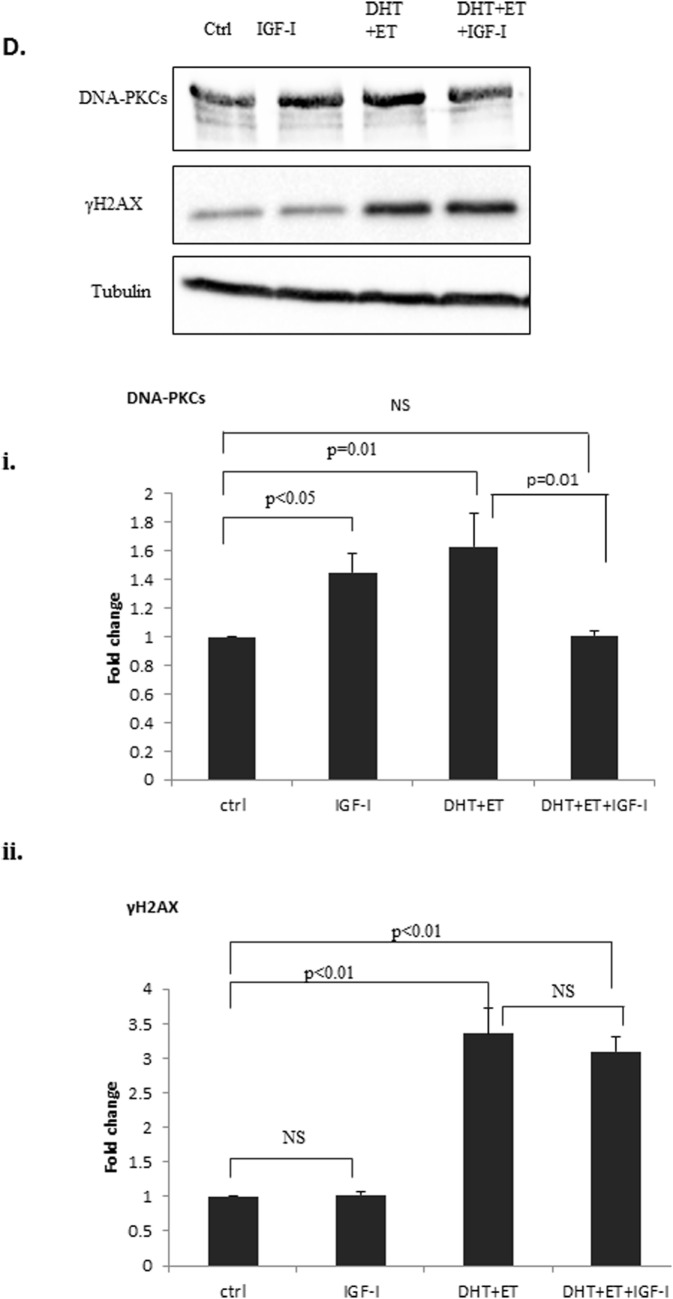
Effects of silencing insulin-like growth factor binding protein-2 (IGFBP-2) or adding insulin-like growth factor-1 (IGF-I) on levels of γH2AX and DNA-dependent protein kinase catalytic subunit (DNAPKcs) in LNCaP prostate cancer cells. Cells were seeded and dosed as in the legend for Figure 1 and following etoposide treatment were incubated in fresh SFM and lysed every 1 h from 3 h in SFM. **(A)** Shows a representative blot of levels of IGFBP-2, γH2AX, DNAPKcs, and β-actin analyzed by Western immunoblotting as described previously (9). **(B)** Densitometry of the western blot shown in **(A)** indicating levels of γH2AX, DNAPKcs normalized to β-actin levels relative to the non-silencing untreated control. Following etoposide, treatment cells were lysed after 4 h in fresh SFM and **(C)** shows a representative western blot that has been repeated three times showing of levels of IGFBP-2, γH2AX, DNAPKcs normalized to tubulin levels. [**(C)**i,ii,iii] densitometry showing the mean changes of three experiments showing levels ofγH2AX and DNAPKcs, respectively, normalized to tubulin levels relative to the non-silencing untreated control. Cells were seeded and dosed with IGF-I (100 ng/ml) as in the legend for Figure 2 and following etoposide treatment cells were lysed after 4 h in fresh SFM. **(D)** Shows a representative western blot that has been repeated three times showing of levels of γH2AX and DNAPKcs normalized to tubulin levels and [**(D)**i,ii] show densitometry of the mean changes of three experiments indicating levels of DNAPKcs and γH2AX, respectively, normalized to tubulin levels.

Figure 3A shows an increase in γH2AX after etoposide treatment, corresponding to a dramatic increase in DSBs. At 3, 4, and 5 h the bands depicting the levels of γH2AX were substantially higher in cells in which IGFBP-2 had been knocked down compared to non-silencing controls. A decrease in the levels of DNAPKcs after IGFBP-2 knockdown was observed at 3, 4, and 5 h after etoposide and DHT dosing compared to non-silencing treated cells. This was verified by densitometry shown in Figure 3B. We repeated the experiment at the 4 h time point to confirm that levels of γH2AX were significantly increased (*p* = 0.003) and those of DNAPKcs were significantly (*p* = 0.011) decreased with IGFBP-2 silenced (*p* = 0.009) (Figure 3Ci,ii,iii). Figure 3Di,ii show that DHT and etoposide treatment alone increased levels of DNAPKcs and of γH2AX (*p* = 0.01 and *p* < 0.01, respectively). Although IGF-I alone increased DNAPKcs (*p* < 0.05), in the presence of DHT and etoposide, IGF-I acted in an opposite way and reduced DNAPKcs (*p* = 0.01). IGF-I, however, had no effect on γH2AX either alone or in combination with DHT and etoposide.

## Discussion

Knowing that high glucose increases the abundance of IGFBP-2 in PCa cells (9), we used this model to assess whether increasing levels of glucose altered the number of TMPRSS2-ERG fusion products induced by exposure to DHT and etoposide and if this was mediated by IGFBP-2. Our data suggest that high glucose increases the number of TMPRSS2:ERG fusion products, and this was not seen when the accompanying increase in IGFBP-2 was prevented, consistent with IGFBP-2 playing a role in this effect. It would be interesting to investigate whether other inducers of IGFBP-2 also elicit such an effect and indeed whether adding exogenous IGFBP-2 to the cells in normo-glycemic conditions also increased the number of TMPRSS2-ERG fusion products induced by exposure to DHT and etoposide, as this would infer a more general role for IGFBP-2. The NHEJ pathway is a process that repairs DSB in DNA (30). Silencing components involved in the process of NHEJ prevents TMPRSS2:ERG gene fusions (25) indicating that NHEJ is a major method for generating fusions. DNAPK is a large protein complex that plays an important role in NHEJ in DNA-DSB repair and possesses a catalytic subunit called DNAPKcs. DNAPK is critical for controlling progression through the cell cycle and maintaining genomic stability (31). As well as DNAPK being modulated through its interactions with DNA, its activity can also be regulated by a variety of other mechanisms, including modulation of DNAPKcs. A study in HeLa cells (32) concluded that DNAPKcs plays an important role in the regulation of γH2AX phosphorylation in response to DNA damage. Phosphorylation of γH2AX is essential to mark the DSB allowing the DNA repair machinery to identify its location (33). Our data suggest that IGFBP-2 has a role in increasing the rate of repair of DSBs by increasing levels of DNAPKcs and this culminates in increased TMPRSS2:ERG gene fusion. An effect of IGFBP-2 on DNAPK has previously been observed: treatment of astrocytes with IGFBP-2 resulted in a direct induction of DNAPKcs (34). Additional studies provide further support suggesting a role of IGFBP-2 in facilitating DNA repair: in glioblastoma studies, IGFBP-2 alters the expression of the following DNA repair genes: X-ray repair complementing defective repair 2, cyclin-dependent kinase inhibitor 1A, and CDC28 protein kinase 2 (35). In addition, a large-scale study, also in glioblastomas, showed that both the DNA-DSB repair pathway and the homologous recombination pathway are associated with IGFBP-2 expression, altering a broad range of proteins including p53, GADD45, TOP2A, and BRCA1 (36). Of all the six similar IGFBPs, IGFBP-2 has most frequently been reported to be overexpressed in a range of human cancers and only IGFBP-2 has been linked to the DNA-DSB repair pathway. It would, however, be interesting to investigate whether other IGFBPs could have similar actions. In our model showing that adding or silencing any of the other IGFBPs had no effect on fusion induction would imply that this was a specific effect of IGFBP-2.

It has become increasingly clear that IGFBPs, including IGFBP-2, can exert effects that are both dependent and independent of its interactions with IGFs (11). To investigate if the effects of IGFBP-2 in promoting TMPRSS2:ERG gene fusions through facilitating DNA repair were dependent on IGF-I, we exposed LNCaP cells to IGF-I alone or following treatment with DHT and etoposide and compared this to the effects of insulin (that does not bind IGFBPs) and EGF (an alternative growth factor). The effects of IGF-I on DNAPKcs might suggest the effects of IGFBP-2 on DNAPKcs were dependent on binding to IGF-I and negating its effect. Further work is required to confirm the IGF-dependency of IGFBP-2 in DSB repair and the induction of fusion; as we did not observe any effect of IGF-I on γH2AX at this time point, although IGF-I induced a reduction in the frequency of fusion products, it was not statistically significant. It is possible that a potential reduction in fusion products induced by IGF-I was not due to an effect on DNA repair but could have been an effect on chromosomal architecture. It has been observed that, in LNCaP-LN3 cells (a derivative of the LNCaP cells that were used in our study), blocking the IGF-I receptor had no effect on γH2AX focus formation, suggesting that activation of the IGF-IR in this cell line has no effect on DSB repair (37).

In summary, our data suggest that exposure to insulin and potentially IGF-I reduced the frequency of formation of fusion products whereas both hyperglycemia and IGFBP-2 increased the number of TMPRSS2-ERG gene fusions and these factors could contribute to the negative impact that obesity has on PCa progression.

## Author Contributions

JH and CP made substantial contributions to the design and with JB, RM, AB were responsible for the work, interpretation, and analysis of the data. All authors contributed to drafting and revising the manuscript and all approved the final version with an agreement of accountability for the work presented.

## Conflict of Interest Statement

The authors declare that there is no conflict of interest that could be perceived as prejudicing the impartiality of the research reported.
